# Measures of co-expression for improved function prediction of long non-coding RNAs

**DOI:** 10.1186/s12859-018-2546-y

**Published:** 2018-12-19

**Authors:** Rezvan Ehsani, Finn Drabløs

**Affiliations:** 1 Department of Mathematics, University of Zabol, Zabol, Iran; 2 Department of Bioinformatics, University of Zabol, Zabol, Iran; 3 Department of Clinical and Molecular Medicine, NTNU - Norwegian University of Science and Technology, NO-7491 Trondheim, Norway

**Keywords:** Function prediction, Gene annotation, Co-expression, Fisher information metric, Sobolev metric, semantic similarity

## Abstract

**Background:**

Almost 16,000 human long non-coding RNA (lncRNA) genes have been identified in the GENCODE project. However, the function of most of them remains to be discovered. The function of lncRNAs and other novel genes can be predicted by identifying significantly enriched annotation terms in already annotated genes that are co-expressed with the lncRNAs. However, such approaches are sensitive to the methods that are used to estimate the level of co-expression.

**Results:**

We have tested and compared two well-known statistical metrics (Pearson and Spearman) and two geometrical metrics (Sobolev and Fisher) for identification of the co-expressed genes, using experimental expression data across 19 normal human tissues. We have also used a benchmarking approach based on semantic similarity to evaluate how well these methods are able to predict annotation terms, using a well-annotated set of protein-coding genes.

**Conclusion:**

This work shows that geometrical metrics, in particular in combination with the statistical metrics, will predict annotation terms more efficiently than traditional approaches. Tests on selected lncRNAs confirm that it is possible to predict the function of these genes given a reliable set of expression data. The software used for this investigation is freely available.

**Electronic supplementary material:**

The online version of this article (10.1186/s12859-018-2546-y) contains supplementary material, which is available to authorized users.

## Background

Long non-coding RNAs (lncRNAs), defined as non-protein-coding transcripts longer than 200 nucleotides, are one of the most common RNA species, but they are in most cases poorly understood with respect to function [1]. It has been shown that lncRNAs play important roles in a wide range of biological process [2, 3] and diseases [4–6]. Possible functions of lncRNAs can be characterized experimentally using gain- and loss-of-function approaches [7, 8], but this is not a straightforward method, for example because lncRNAs can be expressed in multiple isoforms. Therefore, to apply computational methods and algorithms can be a good and accessible supplement to experimental methods for suggesting possible functions of lncRNAs and other un-annotated genes.

Currently, such computational approaches are still at an early stage of development, although its importance has been recognized [9–11]. There are considerable challenges in finding precise and reliable computational approaches due to a lack of suitable data. There is also a lack of databases with relevant features that are suitable for example for machine learning, there is a lack of lncRNAs with known function for training, and for most lncRNAs we are not aware of any common structural features that are important for function. For example, many lncRNA gene sequences are not conserved and do not contain clear motifs [12], which makes it difficult to find and predict the function of lncRNAs by relying on their sequences. A lack of any rich set of molecular interaction data for most lncRNAs is also a limitation with respect to computational annotation [13, 14].

**Fig. 1:**
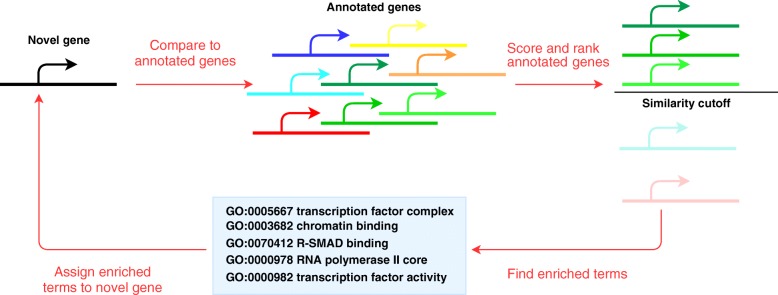
The “guilt by association” principle for prediction of annotation terms for a novel gene. It is based on comparison of gene expression profiles between the novel gene and a set of annotated genes, ranking of the annotated genes according to the similarity of the expression profiles relative to the novel gene, and enrichment analysis of annotation terms in the most highly ranked annotated genes. The significantly enriched terms can be used as an estimate of annotation for the novel gene

One possible and frequently used approach is based on “guilt by association”, i.e., to identify well-annotated genes that seem to be involved in some of the same processes as a given un-annotated gene. This association is often based on co-expression, indicating potential co-regulation of a set of genes [15, 16]. It is then possible to predict function of the un-annotated gene by using information from the well-annotated co-expressed genes, assuming that co-expressed genes may be involved in similar or at least related processes. This approach is illustrated in Fig. 1, and it has been tested in several implementations, with some success. Alam et al. and Gong et al. list several methods and databases for annotation of ncRNAs [17, 18], and most of these use co-expression at some stage, mainly with standard statistical metrics like Pearson or Spearman. For example, Guttman et al. determined several sets of mouse lncRNAs to be related to sets of mRNA for protein-coding genes by Pearson correlation [19], and co-expressed lncRNA-mRNA networks for mouse have also been used by Guo et al. (with “lnc-GFP”) and Liao et al. (using a coding-non-coding (CNC) network computed with Pearson correlation) to assigned function to 340 and 1625 mouse lncRNAs, respectively [20, 21]. Jiang et al. used Pearson correlation to identify co-expressed genes for human lncRNAs (with “LncRNA2Function”), and they could annotate given lncRNAs with significantly enriched gene ontology (GO) terms among the set of co-expressed protein-coding genes, according to the hypergeometric test [22]. Park et al. built a database of lncRNAs (“lncRNAtor”), where they included co-expression by Spearman correlation [23], whereas Zhao et al. developed a web-based application (“Co-LncRNA”) for exploring combinatorial effects of lncRNAs, using linear regression and Spearman correlation to map co-expression between lncRNAs and protein-coding genes [24]. Perron et al. combined co-expression by Pearson correlation with evolutionary conservation in “FuncPred” [25], whereas Zhou et al. used a combination of ChIP-seq, CLIP-seq and RNA-seq (in “lncFunNet”) to predict lncRNA function, using Pearson correlation to estimate co-expression of gene pairs [26]. Zhou et al. also released a toolkit (“lncFunTK”) for calculation of a Functional Information Score (FIS) [27], based on their previous work with lncFunNet. Recently Zhang et al. used a hierarchy of neural networks to predict GO terms for lncRNA genes, implemented as “NeuraNetL2GO”, with Pearson correlation as the measure of co-expression [28].

Thus, most proposed methods rely on identification of co-expressed genes from experimental expression data. Therefore, estimation of co-expression is a crucial step, and relevant alternative metrics should be evaluated. Here we use several metrics for estimating co-expression, in particular statistical (Pearson, Spearman) and geometrical ones (Sobolev, Fisher), and a combination of those. This has been implemented as LNCRNA2GOA, which is available to users. The aim is to provide improved identification of true co-expression. We use an enrichment analysis to identify enriched GO-terms in the co-expressed gene sets, and use this to predict GO terms for the un-annotated gene. We have benchmarked the methods for co-expression on a subset of well-annotated protein-coding genes, using semantic similarity to compare real and predicted GO terms, and also tested the performance on a small number of well-known human lncRNAs.

## Methods

### Data sources

For expression data we have used data from Jiang et al. [22]. They used the information from GENCODE V15 [29] for genomic coordinates and RNA-Seq data of 19 human normal tissues from the Human Body Map 2 project (ArrayExpress accession GSE0554), and read and computed expression values using tophat [30] and cufflinks [31] for all human lncRNA and protein-coding genes. Details are given in the paper by Jiang et al. [22]. GO annotations were downloaded from the Gene Ontology Project [32], and R [33] version 3.3.3, Plyr [34] version 1.8.5, and GOSim [35] version 1.12.0 were used for the implementation.

### Main workflow

The schematic workflow is shown in Additional file 1: Figure S1, where key aspects of this study (i.e., metrics for comparison of expression profiles, and similarity measures for comparing predicted and known annotations) are highlighted. A pseudocode representation of the LNCRNA2GOA algorithm is shown in Table 1. The goal is function prediction for an lncRNA or another poorly annotated gene (denoted as *g*). Let Ϻ be a set of statistical and geometrical methods (see below) and ${Target}_g^m$ be all protein-coding (or well-annotated) genes that are co-expressed with *g* as determined by method *m*. Now the gene *g* will be functionally annotated with significantly enriched annotation terms (here GO) among the set of co-expressed well-annotated protein-coding genes. We use the hypergeometric test to compute the *p*-value of each term *T*:

**Table 1:**
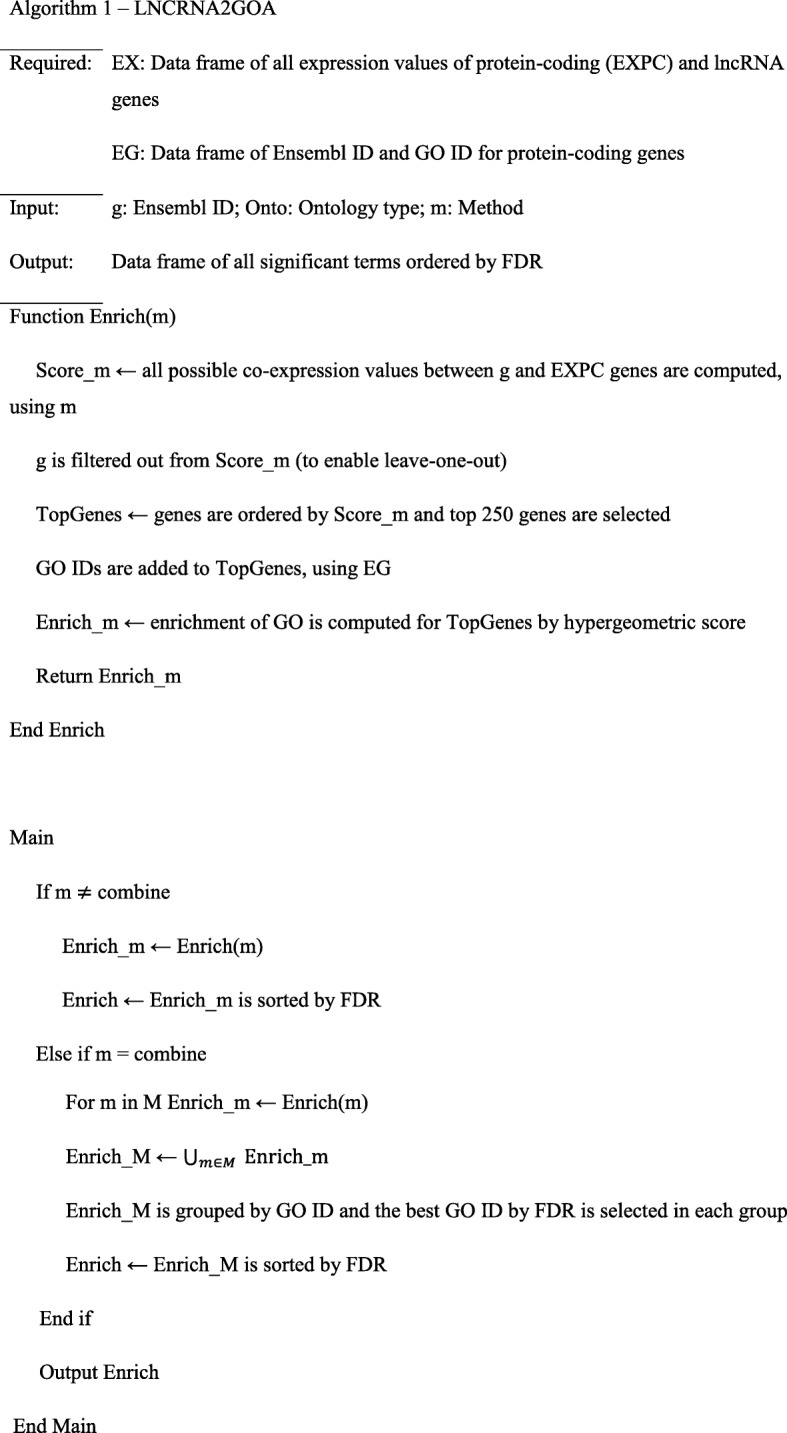
Pseudocode of the LNCRNA2GOA algorithm

1 $$ p=\frac{\sum_{i=t}^{\mathit{\min}\left(n,M\right)}\left(\begin{array}{c}M\\ {}i\end{array}\right)\left(\begin{array}{c}N-M\\ {}n-i\end{array}\right)}{\left(\begin{array}{c}N\\ {}n\end{array}\right)} $$

Herein, *N* is the number of all protein-coding genes, *M* is the number of protein-coding genes that are annotated in the functional term *T*, *n* is the size of ${Target}_g^m$ and *t* is the number of genes in ${Target}_g^m$ that are annotated in the functional term *T*. Because the statistical analysis is not appropriate for problems of small size, we exclude GO terms with less than five annotated protein-coding genes from the enrichment analysis, as recommended by Jiang et al. [22]. We also use false discovery rate (FDR) for correction for multiple hypothesis tests. The significance cut-off of corrected *p*-value is set as 0.05.

### Methods to identify ${\mathrm{Target}}_{\mathrm{g}}^{\mathrm{m}}$

To identify co-expressed genes for a typical gene *g*, we used the geometrical metrics *Sobolev* and *Fisher information*, in addition to the statistical metrics Pearson and Spearman.

#### Sobolev metric

In this section, we use definitions and notations as in [36]. We start with the usual p-inner product. Let *f, g* be real-valued functions (in this case *f* and *g* values are the expression vectors of two genes *f* and *g*):

2 $$ {\left\langle f,g\right\rangle}_p={\left(\sum \limits_{k=1}^n{\left|{f}_k.{g}_k\right|}^p\right)}^{\frac{1}{p}} $$

By this notation, Sobolev inner product, norm and meter of degree *k* respectively can be defined by:

3 $$ {\left\langle f,g\right\rangle}_{p,\alpha}^S={\left\langle f,g\right\rangle}_p+\alpha {\left\langle {D}^kf,{D}^kg\right\rangle}_p $$

4 $$ {\left\Vert f\right\Vert}_{p,k,\alpha}^S=\sqrt{{\left\langle f,f\right\rangle}_{p,\alpha}^S} $$

5 $$ {d}_{p,k,\alpha}^S\left(f,g\right)={\left\Vert f-g\right\Vert}_{p,k,\alpha}^S $$

where *D*^*k*^ is the *k*th differential operator. For the special case *p* = 2 and *α* = 1 an interesting connection to the Fourier-transform of analysis can be made; let $\widehat{f}$ be the Fourier-transform *f*

6 $$ \widehat{f}\left({\omega}_k\right)=\sum \limits_{j=1}^{N-1}{g}_j\exp \left(-i\frac{2\pi kj}{N}\right) $$

where ${\omega}_k=\frac{2\pi k}{N}$ and $i=\sqrt{-1}$. Finally, the norm can be written as

7 $$ {\left\Vert f\right\Vert}_{2,k,1}^S=\sqrt{\sum \limits_{j=1}^{N-1}{\left(1+{\omega}_j\right)}^k{\left|\widehat{f}\left({\omega}_j\right)\right|}^2} $$

In this work we used metric (5) with norm (7) and *k = 1*.

#### Fisher information metric

In this section we use definitions and notations as in [37]. To define Fisher information metric we first introduce the *n*-simplex *P*_*n*_ defined by

8 $$ {P}_n=\left\{x\in {R}^{n+1}:\forall i,{x}_n\ge 0,\sum \limits_{i=1}^{n+1}{x}_i=1\right\} $$

The coordinates {*x*_*i*_} describe the probability of observing different outcomes in a single experiment (or expression value of a gene in *i*th cell type). The Fisher information metric on *P*_*n*_ can be defined by

9 $$ {J}_{ij}=\sum \limits_{k=1}^{n+1}\frac{1}{x_k}\frac{\partial {x}_k}{\partial {x}_i}\frac{\partial {x}_k}{\partial {x}_j} $$

We now define a well-known representation of the Fisher information as a pull-back metric from the positive *n*-sphere ${S}_n^{+}$;

10 $$ {S}_n^{+}=\left\{x\in {R}^n;\forall i,{x}_n\ge 0,\sum \limits_{i=1}^{n+1}{x}^2=1\right\} $$

The transformation $T:{P}_n\to {S}_n^{+}$ defined by

11 $$ T(x)=\left(\sqrt{x_1},\dots, \sqrt{x_{n+1}}\right) $$

pulls back the Euclidean metric on the surface of the sphere to the Fisher information on the multinomial simplex. Actually, the geodesic distance for *x*, *y* ∈ *P*_*n*_ under the Fisher information metric may be defined by measuring the length of the great circle on ${S}_n^{+}$ between *T*(*x*) and *T*(*y*)

12 $$ d\left(x,y\right)=\mathrm{acos}\left(\sum \limits_{i=1}^{n+1}\sqrt{x_i{y}_i}\right) $$

#### Size of ${Target}_g^m$

In the previous section we introduced four methods to measure similarity between genes based on their expression values, which can be used to rank genes. Now the challenge is to determine a threshold for identifying the most informative set of correlated genes as ${Target}_g^m$*.* Some studies, for example [22] used a threshold of 0.9 on the Pearson metric, but this does not identify an optimal cutoff; sometimes it returns thousands of co-regulated genes, and sometimes nothing. It is also difficult to set a threshold in a similar way when using a geometrical metric. Therefore, we tried to select a threshold based on a possible number of ${Target}_g^m$ elements. We selected a range of different sizes for subsets of the most similar genes from the ${Target}_g^m$ set, including {50 ∗ *x*| *x* = 1, 2, …, 10}, and analysed how well the algorithm could predict some well-known lncRNAs. This analysis showed that a selection of the top 250 co-expressed protein-coding genes seemed to have optimal performance for prediction of GO terms for some well-known lncRNAs.

#### Combination of methods

Let ${SigGOs}_g^{m,r}={\left[{SigGOs}_g^m\right]}^r$ be the *r* most significant terms assigned to gene *g* by method *m*. Since the optimal method *m* is individually different we can assign ${SigGOs}_g^{r,s}={\left[{\cup}_{{}_{m\in \mathrm{M}}{SigGOs}_g^{m,r}}\right]}^s$ to the gene, and this will increase the accuracy of predictions (see Results and discussion). If there are identical terms for some methods, we just consider the term with the minimum corrected *p*-value. Actually, in the combination method the algorithm first collects the *r* most significant terms for all *m* ∈ Μ, then selects the *s* most significant terms from this collection. When *r* = all and *s* = all then the algorithm returns all significant terms.

### Evaluation on protein-coding genes

Before evaluating the performance of our method on some known lncRNA genes, we benchmarked it on a set of well-annotated genes. Since protein-coding genes in general are much more well-annotated than lncRNA genes, this set was based on protein-coding genes. We selected protein-coding genes annotated by 5 or 6 molecular function (MF) terms and 9 or 10 biological process (BP) terms in the GO database. This represents the average number of GO-terms in each set, as the genes were on average annotated by 4.67 MF and 9.25 BP terms. Therefore the benchmark set has a typical (average) level of annotation. It consists of 352 protein-coding genes (denoted as Test_352_), and is available as part of the LNCRNA2GOA distribution [38]. We used a “leave-one-out” approach for the actual benchmarking. That is, for each gene in Test_352_ we treated the gene as unannotated and predicted GO terms for the gene by each of five different methods (Pearson, Spearman, Sobolev, Fisher, and Combine, which is a combination of all four methods). We then used the TopoICSim [39] and GOSemSim [40] approaches to estimate similarity between real and predicted GO annotations for each gene in Test_352_. TopoICSim and GOSemSim are two algorithms for measuring semantic similarity between pairs of genes. To have a more realistic semantic measure for benchmarking, we restricted the size of the output set of enriched terms by selecting ${SigGOs}_g^{\left(m,2\times L\right)}$ where *L* is the number of GO terms for gene *g* in Test_352_ based on the gene ontology database. For the Combine case, where we find an optimal combination of predictions from each of the four different methods, we evaluated the performance first for ${SigGOs}_g^{\mathrm{M},2\times L,2\times L}$ (*Combine_2L*) and then For ${SigGOs}_g^{\mathrm{M},2\times L,4\times L}$ (*Combine_4L*).

## Results

### Evaluation on Test_352_

**Fig. 2:**
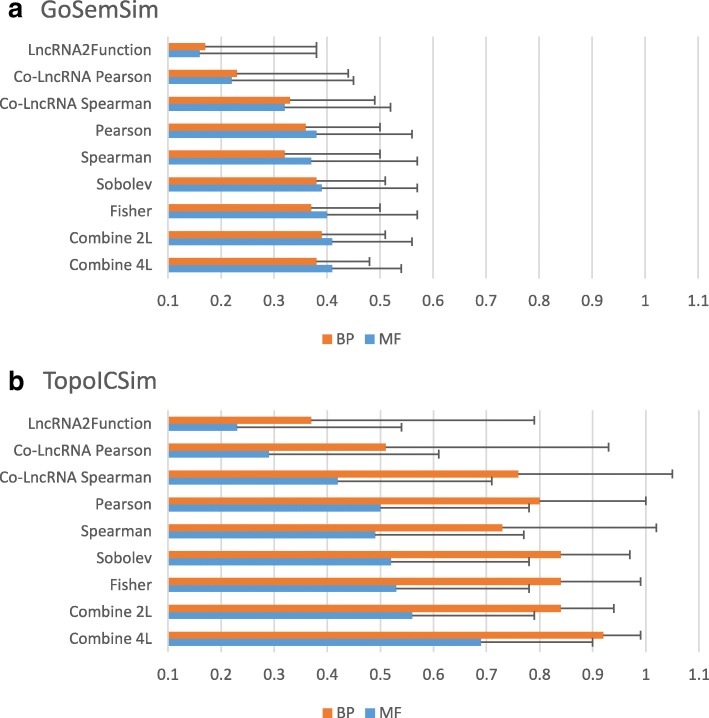
Evaluation of the different similarity metrics for 352 well-annotated protein-coding genes (Test_352_). For each gene in Test_352_ all methods were applied for prediction of function, and similarity between real and predicted terms were measured with TopoICSim and GOSemSim. The table shows average similarity scores over the test set, with standard deviation. LncRNA2Function: Co-expressed protein-coding genes was obtained for each gene in Test_352_ using [22] with Pearson correlation coefficient > 0.9. Co-LncRNA_Pearson or _Spearman: Co-expressed protein-coding genes was obtained for each gene in Test_352_ using [24] with Pearson or Spearman correlation coefficient > 0.8 and LncRNA2Function expression data

Before applying LNCRNA2GOA to human lncRNAs, we first benchmarked the performance on the well- annotated set of protein-coding genes, Test_352_. We have applied all metrics in Ϻ for each gene in Test_352_ and evaluated semantic similarity between actual and predicted annotation as measured by the GOSemSim and TopoICSim methods. We also included results for LncRNA2Function [22] and Co-LncRNA [24]. The results for Co-LncRNA were based on the published computational approach, but using the LncRNA2Function database, to keep the results comparable. The average semantic similarities for each method and metric are shown in Fig. 2. In all cases the TopoICSim measure shows better performance than GOSemSim. It has previously been shown that TopoICSim seems to be a more correct measure for semantic similarity compared to other approaches [39], therefore this most likely reflects a real similarity in predicted annotation, and not a systematic bias in measurements. In particular for TopoICSim there is a good correlation between performance in MF and BP, indicating that the improved performance is not random, but due to the choice of better methods. The results also show that the geometrical metrics predict function more efficiently than statistical metrics, and in particular the combined measure shows quite good performance. It can be argued that the standard deviation (SD) of the averages is high, indicating a variation in performance. However, the SD is lower for the Combine measure. The overall performance seems to be better for BP compared to MF. It is possible that this reflects a bias in the dataset, as each gene in *Test*_352_ has almost twice as many BP terms compared to MF terms. In summary, Fig. 2 shows that the combined approach has good performance with respect to prediction of GO terms, in particular for terms related to BP.

### Evaluation on data from FuncPred

**Fig. 3:**
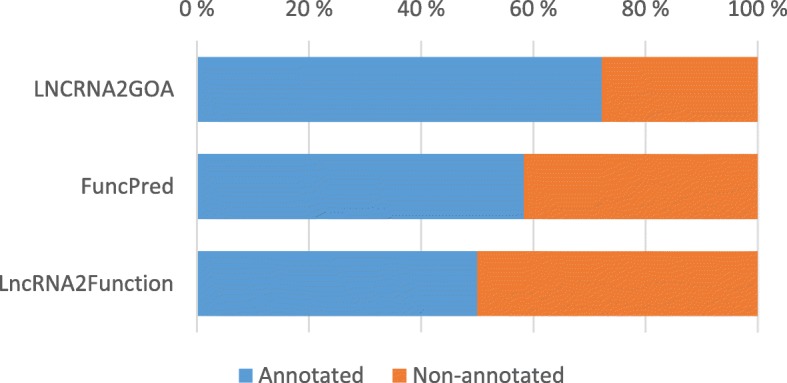
GO terms for a manually annotated set of 37 lncRNAs from FuncPred has been predicted using LNCRNA2GOA, FuncPred and LncRNA2Function, and the number of successful predictions has been counted

We also tested the performance of LNCRNA2GOA on a set of 37 manually annotated lncRNAs from FuncPred [25]. Figure 3 shows the number of successful predictions by LNCRNA2GOA, FuncPred and LncRNA2Function [22]. Here a prediction was counted as successful if at least one of the correct GO terms could be predicted. The same approach has previously been used for example in testing of the NeuraNetL2GO method [28]. Again we see that the best performance was achieved with the LNCRNA2GOA approach.

### Comparison to functional predictions by lncFunTK

Finally, we have done a qualitative evaluation of predictions performed with LNCRNA2GOA, compared to predictions by lncFunTK with data from HeLa cells and Human Body Map, provided as Table S2 in Zhou et al. [27]. The lncFunTK predictions are sorted according to Functional Information Score (FIS), which is a predicted functional importance of a given lncRNA, based on a combination of several data types (ChIP-seq, CLIP-seq, RNA-seq). We have focussed on the 100 most significant cases from lncFunTK according to FIS, and used LNCRNA2GOA to predict GO terms for 50 of those genes where Ensembl IDs could reliably be assigned (see Additional file 2: Table S1). The predicted GO terms were then compared to the GO terms from lncFunTK, using experimental data from literature as a common reference. We were able to retrieve PubMed entries based on gene name for only 20 of the 50 genes, which limits the comparison. However, for more than half of these genes the predictions from LNCRNA2GOA showed at least some similarity to literature data (see Additional file 3: Table S2). For lncFunTK, on the other hand, the available predictions consist of only a single GO term per gene, and most of these terms are identical. For the set of 50 genes, 35 entries are classified as GO:0045944 (positive regulation of transcription from RNA polymerase II promoter) by lncFunTK, and this strong preference for GO:0045944 is the same for the full set of predictions.

**Table 2:** Summary information describing the five case studies for human lncRNAs

Ensembl ID	Gene symbol	Name Function	Selected references
ENSG00000228630	HOTAIR	Hox transcript antisense RNA Development process and morphogenesis, cancer metastasis and invasiveness	[41, 42]
ENSG00000206337	HCP5	HLA complex P5 Immune responses	[43, 44]
ENSG00000251164	HULC	Heptacellular carcinoma up-regulated long non-coding RNA Liver cancer and colorectal carcinomas that metastasize to the livers	[45, 46]
ENSG00000130600	H19	Imprinted maternally expressed transcript Infertility and multiple cancers such as breast, cervical, liver and bladder	[47, 48]
ENSG00000225937	PCA3	Prostate cancer associated 3 Prostate cancer	[49, 50]

It is difficult to do a direct comparison of predictions from these two methods. Both use RNA-seq data from multiple tissue types to estimate co-expression. However, the lncFunTK approach also ranks genes using additional data (in this case, on HeLa cells), which may give some preference for properties that are particularly enriched in this cell type (for example increased transcription). LNCRNA2GOA may to a larger extent display properties that are important across the full range of activities where each lncRNA is involved. This may explain some of the differences in the output between lncFunTK and LNCRNA2GOA. However, it is our general impression that the predictions from LNCRNA2GOA contain more information, compared to lncFunTK, and that several of these predictions seem to be consistent with observations from literature.

### Functional annotation of human lncRNAs

There is a lack of good “gold standard” datasets for human lncRNAs with known function that can be used for benchmarking. However, we have used a small set of five well-known lncRNAs from literature as examples to show the efficiency of LNCRNA2GOA. These lncRNAs have previously been used for documenting the performance of in particular LncRNA2Function [22]. The summary information describing these example lncRNAs, their functions and references are shown in Table 2.

To examine whether LNCRNA2GOA is able to functionally annotate the lncRNAs that are listed in Table 2, we applied the algorithm with defaults (i.e., ontology set as Biological Process and using the Combine method with (${SigGOs}_g^{\mathrm{M}, all, all}$), and prediction results are presented and discussed for each lncRNA separately. The top 10 predictions are in each case are given in Table 3.

**Table 3:** Top 10 biological processes assigned to each of the selected case studies

GO ID	*P*-value	FDR	Term
HOTAIR (development and morpogenesis)			
GO:0032964	0.00e+ 00	0.00e+ 00	collagen biosynthetic process
GO:0030199	1.72e-14	9.36e-12	collagen fibril organization
GO:0030198	1.39e-09	3.98e-07	extracellular matrix organization
GO:0007275	2.07e-07	6.27e-05	multicellular organism development
GO:0035115	5.31e-07	7.63e-05	embryonic forelimb morphogenesis
GO:0060272	8.21e-07	1.00e-04	embryonic skeletal joint morphogenesis
GO:0048704	7.01e-07	1.49e-04	embryonic skeletal system morphogenesis
GO:0001568	2.13e-06	1.88e-04	blood vessel development
GO:0007506	7.72e-06	1.00e-03	gonadal mesoderm development
GO:0002063	1.58e-05	1.01e-03	chondrocyte development
HCP5 (immune- and AIDS-related processes)			
GO:0002480	0.00e+ 00	0.00e+ 00	antigen processing and presentation of exogenous peptide antigen via MHC class I, TAP-independent
GO:0002504	0.00e+ 00	0.00e+ 00	antigen processing and presentation of peptide or polysaccharide antigen via MHC class II
GO:0002376	1.35e-55	3.37e-53	immune system process
GO:0050776	1.10e-53	1.22e-50	regulation of immune response
GO:0006955	6.98e-50	1.39e-47	immune response
GO:0002250	6.06e-28	1.35e-25	adaptive immune response
GO:0050852	3.56e-27	5.93e-25	T cell receptor signaling pathway
GO:0019882	7.67e-27	1.09e-24	antigen processing and presentation
GO:0045087	6.77e-23	1.26e-20	innate immune response
GO:0042110	8.62e-20	1.21e-17	T cell activation
HULC (liver-related processes)			
GO:0002933	0.00e+ 00	0.00e+ 00	lipid hydroxylation
GO:0006547	0.00e+ 00	0.00e+ 00	histidine metabolic process
GO:0006572	0.00e+ 00	0.00e+ 00	tyrosine catabolic process
GO:0010873	0.00e+ 00	0.00e+ 00	positive regulation of cholesterol esterification
GO:0010898	0.00e+ 00	0.00e+ 00	positive regulation of triglyceride catabolic process
GO:0016098	0.00e+ 00	0.00e+ 00	monoterpenoid metabolic process
GO:0030300	0.00e+ 00	0.00e+ 00	regulation of intestinal cholesterol absorption
GO:0034371	0.00e+ 00	0.00e+ 00	chylomicron remodelling
GO:0034378	0.00e+ 00	0.00e+ 00	chylomicron assembly
GO:0042737	0.00e+ 00	0.00e+ 00	drug catabolic process
H19 (cancer-related processes)			
GO:0007565	3.46e-14	2.79e-11	female pregnancy
GO:0060397	7.14e-08	1.92e-05	JAK-STAT cascade involved in growth hormone signaling pathway
GO:0070234	1.95e-06	3.93e-04	positive regulation of T cell apoptotic process
GO:0007292	3.90e-05	3.50e-03	female gamete generation
GO:0016486	3.90e-05	3.50e-03	peptide hormone processing
GO:0030325	3.90e-05	3.50e-03	adrenal gland development
GO:0007267	5.08e-05	4.31e-03	cell-cell signalling
GO:0042060	8.28e-05	5.50e-03	wound healing
GO:0006703	1.55e-04	8.91e-03	estrogen biosynthetic process
GO:0030540	4.66e-04	1.75e-02	female genitalia development

#### HOTAIR

HOTAIR (*Hox transcript antisense RNA*) is an lncRNA known to be involved in development, cancer and high risk metastases, at least partly through interaction with PRC2 and regulation of HOX genes [41, 42]. To investigate whether LNCRNA2GOA is able to associate functionally relevant GO terms with HOTAIR, we applied the algorithm with defaults parameters. This identified 124 GO BP terms in total. As expected, most significantly enriched terms are associated with development and morphogenesis, and cell metastasis. The results in Table 3 indicate that LNCRNA2GOA successfully identifies functionally relevant GO terms for HOTAIR.

#### HCP5

HCP5 (*HLA complex P5*) is an lncRNA associated with immune response, and it is associated with for example AIDS [43] and virus-related cancers [44]. When testing whether HCP5 can be annotated by LNCRNA2GOA, we found that HCP5 was associated with 333 GO terms for biological processes. As expected, most of them involve the immune system and immune response, and functional terms that are associated with the development of AIDS (see Table 3). A relevant example is regulation of immune response (GO:0050776).

#### HULC

HULC (*Heptacellular carcinoma up-regulated long non-coding RNA*) is known to be upregulated in liver cancer and associated with tumorigenesis [45, 46]. We used LNCRNA2GOA to assess whether HULC can be correctly assigned to have liver-related functions. The enrichment output showed 177 GO terms for biological processes associated with HULC. Most of these terms are involved in functions related to liver and lipids, such as lipid hydroxylation (GO:0002933) and chylomicron remodelling (GO:0034371). Table 3 shows the top 10 GO functional terms enriched in protein-coding genes that are co-expressed with the liver-related lncRNA HULC.

#### H19

H19 (*Imprinted maternally expressed transcript*) is known to be important for fertility and several processes associated with female disease risk, including cancer [47, 48]. LNCRNA2GOA identified 53 GO terms for biological processes as associated with H19. Several of these terms suggested strongly that H19 can play an important role in infertility or breast cancer, such as female pregnancy (GO:0007565), female gamete generation (GO:0007292), and adrenal gland development (GO:0030325). There were also other relevant GO terms, as for example, JAK-STAT cascade involved in growth hormone signalling pathway (GO:0007565), cell-cell signalling (GO:0007267), and cell proliferation (GO:0008283). This indicates that H19 can play a role in various cancers and other conditions where JAK-STAT signalling is important. The top 10 GO terms for biological processes are shown in Table 3.

#### PCA3

PCA3 (*Prostate cancer associated 3*) is strongly upregulated in prostate cancer [49, 50]. LNCRNA2GOA identified 25 terms for GO biological processes as associated with PCA3. There were three terms directly involved in prostate cancer; urinary bladder development (GO:0060157), prostate gland development (GO:0030850), and prostate epithelial cord arborization involved in prostate glandular acinus morphogenesis (GO:0060527). PCA3 is a quite challenging case, but this prediction seems to be an improvement over a previous prediction by LncRNA2Function, where only a single pathway linked to androgen receptor was identified, and in some aspects also predictions by the more recent FARNA tool [17].

#### Additional tests

We also tested LNCRNA2GOA on a few cases not included in the original LncRNA2Function test set, and got similar results. The two most significant GO BP terms for MALAT1 (*Metastasis associated lung adenocarcinoma transcript 1*) were ion transport (GO:0006811) and excretion (GO:0007588), which seems to be consistent with the observation that MALAT1 often is associated with kidney function and with renal cell carcinoma [51]. For LINC00152, also known as CYTOR (*Cytoskeleton regulator RNA*), the two most significant terms were cell adhesion (GO:0007155) and extracellular matrix organisation (GO:0030198), which is consistent with the observation that this lncRNA influences the properties of breast cancer cells with respect to for example invasion and migration [52]. For LINC-ROR (*Long intergenic non-protein coding RNA, regulator of reprogramming*) the two most significant terms were chromatin silencing at rDNA (GO:0000183) and nucleosome assembly (GO:0006334), which may be consistent with observations that LINC-ROR is involved in regulation of differentiation of embryonic stem cells [53].

We also tested the performance on a more general data set, using a set of prostate cancer-associated lncRNAs with unknown molecular mechanism, published by Mitobe et al. [54]. Most of these lncRNAs are upregulated in prostate cancer, and it has been shown that RNA interference (RNAi) towards these lncRANs leads to a reduction in proliferation, making it reasonable to assume that their upregulation in prostate cancer contributes to increased proliferation in cancer tissue. However, function prediction on this gene set illustrates one of the main challenges. The lack of proper benchmark data, and the fact that the molecular mechanism for these lncRNAs is unknown, makes it difficult to assess the quality of the predictions. There are some cases where recent results seem to support some of the predictions. For example, for SNHG1 (*Small nucleolar RNA host gene 1*) a highly significant prediction is for positive regulation of histone H3-K27 methylation (GO:0061087), which is consistent with recent results from Yu et al. [55] showing that SNHG1 may be involved in epigenetic silencing of the tumour suppressor CDKN1A. However, in most cases the predictions need further verification. The prediction output is therefore included here as supplementary material for future assessment (see Additional file 4: Table S3).

## Discussion

In recent years, thousands of lncRNAs have been discovered that probably play important roles in many different biological processes and diseases, but unfortunately the vast majority of them still need to be functionally annotated. In this paper, we present an improved approach for estimating co-expression for computational function prediction. We compare several measures for estimating co-expression, covering both statistical and geometrical ones, and this gives improved identification of true co-expression. We use an enrichment analysis to identify enriched GO terms in the co-expressed gene set, and use this to predict GO terms for un-annotated genes. This can be any un-annotated gene, but here in particular human lncRNAs.

We have benchmarked the co-expression for enrichment analysis on a subset of well-annotated protein-coding genes. For each gene the GO terms were predicted (without using the known terms for the gene), and the fit between predicted and known GO terms was measured using semantic similarity measures. This showed good correlation between predicted and known GO terms, in particular for terms related to biological process (BP) and when using a combined similarity measure on gene expression. These score values are clearly better than the score values achieved using Pearson or Spearman, which previously has been the most common approach. The procedure was then tested on lncRNAs, in particular using a set of five well-described lncRNAs tested in previous publications [22]. The predicted GO-terms showed good correspondence with published functional descriptions of these lncRNAs. This shows that it is possible to predict the function of both protein-coding and ncRNA genes, given a reliable set of expression data.

There is still room for improvement. Although the prediction is successful in many cases (indicated by the high average similarity score in the benchmarking), the high SD indicates that there are specific cases where the prediction is less successful. It would be very useful if we were able to identify and focus on cases where prediction is most likely to be successful. The performance may also be sensitive to the quality, variation and annotation of the reference data. The approach used for enrichment analysis will also influence the result.

## Conclusion

The results presented here show that approaches for computational gene annotation based on co-expressed genes can provide useful annotations, in particular when using improved estimates of co-expression based on a combination of geometrical and statistical metrics.

## Additional files

Additional file 1: **Figure S1.** A flowchart illustrating the approach used for prediction and benchmarking in LNCRNA2GOA. (PDF 342 kb)

Additional file 2: **Table S1.** Predicted annotation for a set of lncRNAs from HeLa, previously analysed with lncFunTK by Zhou et al. [27] and listed in their Table S2. (TXT 76 kb)

Additional file 3: **Table S2.** Predicted annotations from Additional file 3: Table S1 for lncRNAs found in PubMed, illustrated with selected PubMed references. (TXT 435 kb)

Additional file 4: **Table S3.** Predicted annotation for a set of lncRNAs with unknown mechanism as described by Mitobe et al. [54] in their Table 1 and discussed in the main paper. (PDF 475 kb)

## Abbreviations

(l)ncRNA: (long) non-coding RNA
BP: Biological process
GO: Gene ontology
MF: Molecular function
SD: Standard deviation
