# Supplementary material for: Measures of co-expression for improved function prediction of long non-coding RNAs
Source: BMC Bioinformatics. 2018 Dec 19;19:533. doi: 10.1186/s12859-018-2546-y (PMC6300029; doi:10.1186/s12859-018-2546-y)
Supplement: Supplementary file 1 — Figure S1. A flowchart illustrating the approach used for prediction and benchmarking in LNCRNA2GOA. (PDF 342 kb) [file 12859_2018_2546_MOESM1_ESM.pdf]

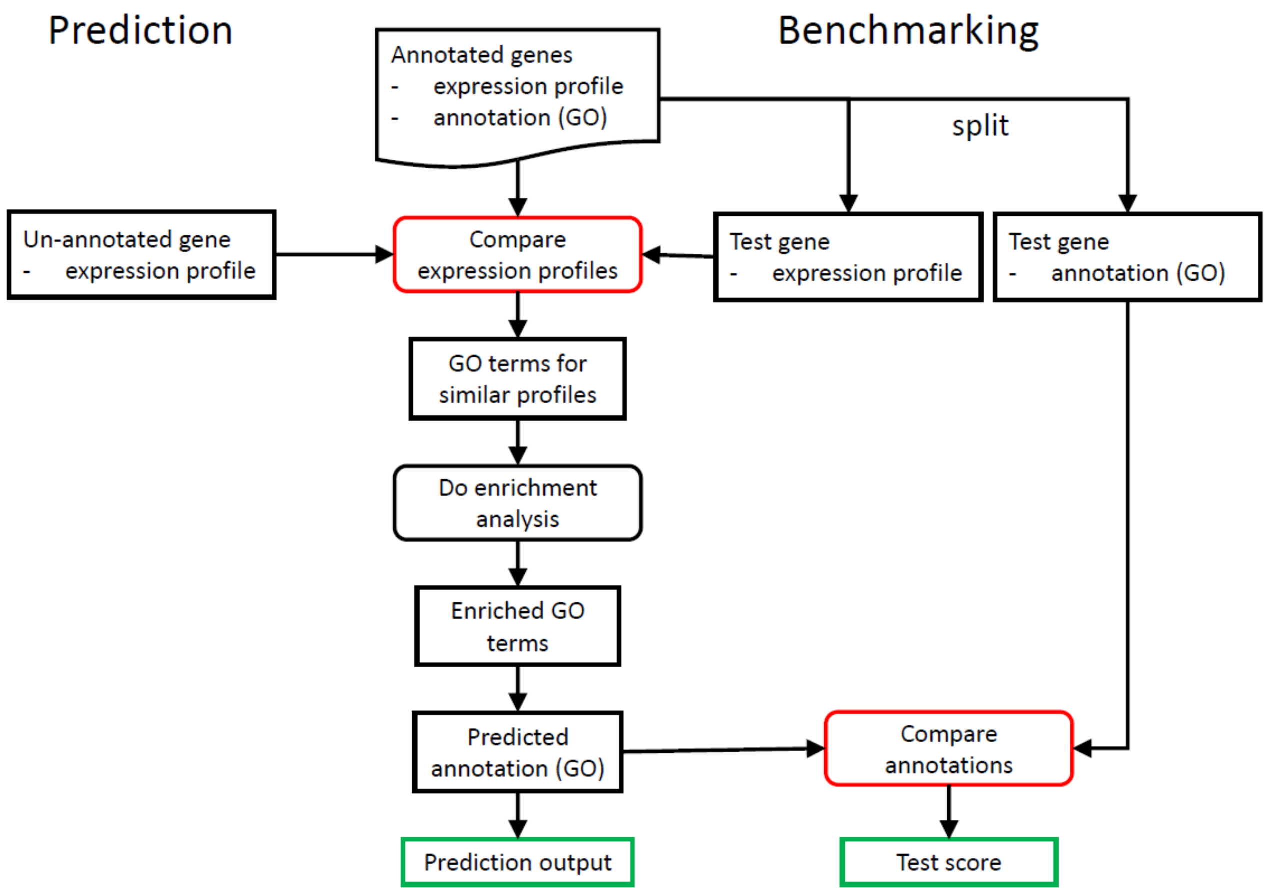

Figure S1 The principle used in LNCRNA2GOA for prediction of annotation terms, based on comparison of expression profiles ("Compare expression profiles"), with introduction of novel similarity measures, and how this prediction is benchmarked by doing a semantic comparison of real and predicted annotation terms ("Compare annotations").
