# Supplementary material for: Measures of co-expression for improved function prediction of long non-coding RNAs
Source: BMC Bioinformatics. 2018 Dec 19;19:533. doi: 10.1186/s12859-018-2546-y (PMC6300029; doi:10.1186/s12859-018-2546-y)
Supplement: Supplementary file 4 — Table S3. Predicted annotation for a set of lncRNAs with unknown mechanism as described by Mitobe et al. [54] in their Table 1 and discussed in the main paper. (PDF 475 kb) [file 12859_2018_2546_MOESM4_ESM.pdf]

Table S2 – Predicted GO terms for genes with PubMed references

Case[1]	*Gene*	*lncFunTK prediction*[2]	*LNCRNA2GOA predictions (selected)*[3] *GO_ID FDR GO_term*	*PubMed ID*[4]	*Comment (based on literature)*
4	MALAT1<br>ENSG00000251562	GO:0045944	1 GO:0006811 2.16e-12<br>ion transport<br>2 GO:0007588 2.36e-10<br>excretion	29781038<br>(735 refs)	MALAT1 is associated with renal function
14	B4GALT1-AS1<br>ENSG00000233554	GO:0045944	1 GO:0007283 1.27e-05<br>spermatogenesis<br>2 GO:0051321 1.27e-05<br>meiotic cell cycle	30182452<br>29382663<br>26315939	B4GALT1-AS1 reqruits HuR, which is essential for spermatogenesis (PMID 21737689)
17	NEAT1<br>ENSG00000245532	GO:0000122	1 GO:0006590 0.00e+00<br>thyroid hormone generation	28000845<br>(309 refs)	NEAT1 promotes malignant progression of thyroid carcinoma
21	LOC648987<br>ENSG00000177738	GO:0045944	1 GO:0007049 2.15e-19<br>cell cycle<br>2 GO:0051301 1.00e-14<br>cell division	27782156	LOC648987 is hyper-methylated in metastases of lung adenocarcinoma
24	LINC01600<br>ENSG00000164385	GO:0045944	1 GO:0000398 2.21e-04<br>mRNA splicing, via spliceosome<br>2 GO:0030030 2.21e-04<br>cell projection organization	27903974	-
30	PXN-AS1<br>ENSG00000255857	GO:0045944	1 GO:0031118 0.00e+00<br>rRNA pseudouridine synthesis<br>7 GO:0000380 1.95e-05<br>alternative mRNA splicing, via spliceosome	28553938	MBNL3 splicing factor promotes hepatocellular carcinoma through alternative splicing of lncRNA-PXN-AS1
38	TOB1-AS1<br>ENSG00000229980	GO:0045944	1 GO:0016567 7.21e-04<br>protein ubiquitination<br>2 GO:0006004 7.85e-03<br>fucose metabolic process	30210918	-
50	LOC284454<br>ENSG00000267519	GO:0045944	1 GO:0007565 2.94e-11<br>female pregnancy<br>5 GO:0060397 2.02e-05<br>JAK-STAT cascade involved in growth hormone signaling pathway	29227193<br>30380023	A functional role of LOC284454 in breast cancer pathobiology is suggested

52	LINC00592<br>ENSG00000258279	GO:0045944	2 GO:0007283 8.46e-25<br>spermatogenesis<br>9 GO:0030154 9.53e-08<br>cell differentiation	30362566	LINC00592 may bind miR-449a and miR-34a-5p, and both have previously been identified as differentiation-inducing microRNAs (PMID 27764804)
53	MIR22HG<br>ENSG00000186594	GO:0045944	1 GO:0022617 4.97e-05<br>extracellular matrix disassembly<br>2 GO:0030198 4.97e-05<br>extracellular matrix organization	(13 refs)	-
56	CASC11<br>ENSG00000249375	GO:0045944	1 GO:0007186 7.30e-08<br>G-protein coupled receptor signaling pathway<br>2 GO:0050911 7.29e-04<br>detection of chemical stimulus involved in sensory perception of smell	30200804<br>28761083<br>27012187	-
57	FLJ22447<br>ENSG00000232774	GO:0045944	1 GO:0006281 4.76e-05<br>DNA repair<br>2 GO:0006260 1.81e-04<br>DNA replication	29346528	-
59	LINC00324<br>ENSG00000178977	GO:0045944	5 GO:0002376 4.73e-37<br>immune system process<br>6 GO:0006955 2.78e-26<br>immune response<br>7 GO:0002250 4.72e-24<br>adaptive immune response	30146820<br>29915327<br>27373735<br>26780889	LINC00324 can be used for predicting prognosis among patients with thymoma
69	SBF2-AS1<br>ENSG00000246273	GO:0045944	1 GO:0006334 3.48e-14<br>nucleosome assembly<br>4 GO:0045814 8.80e-10<br>negative regulation of gene expression, epigenetic	27154193<br>(7 refs)	SBF2-AS1 promotes proliferation in non-small cell lung cancer, could bind with core component of polycomb repressive complex
71	BDNF-AS<br>ENSG00000245573	GO:0045944	2 GO:1904037 0.00e+00<br>positive regulation of epithelial cell apoptotic process<br>6 GO:0007399 1.38e-03<br>nervous system development	27935942<br>(330 refs)	Inhibition of BDNF-AS provides neuroprotection against ischemic injury

77	HOXC-AS3<br>ENSG00000251151	GO:0006366	1 GO:0009952 2.79e-17<br>anterior/posterior pattern specification<br>6 GO:0048704 1.21e-08<br>embryonic skeletal system morphogenesis	30353595<br>30286788	HOXC10 regulates osteogenesis of mesenchymal cells through interaction with lncHOXC-AS3
81	LIFR-AS1<br>ENSG00000244968	GO:0045944	1 GO:0000042 1.34e-02<br>protein targeting to Golgi<br>2 GO:0046907 1.34e-02<br>intracellular transport	29807108<br>26979631<br>26725846<br>26417400	-
82	LINC00319<br>ENSG00000188660	GO:0045944	1 GO:0044332 0.00e+00<br>Wnt signaling pathway involved in dorsal/ventral axis specification<br>2 GO:0008544 8.98e-11<br>epidermis development	28800794<br>30243935<br>30145798<br>29408583	linc00319 promotes cell proliferation and invasion in lung cancer cells
104	ZFAS1<br>ENSG00000177410	GO:0000122	2 GO:0070383 0.00e+00<br>DNA cytosine deamination<br>9 GO:0002376 2.97e-17<br>immune system process<br>13 GO:0051607 1.36e-09<br>defense response to virus	28825667<br>(58 refs)	Micropeptides from ZFAS1 are differentially regulated in viral infection
105	LINC-ROR<br>ENSG00000258609	GO:0007186	1 GO:0000183 1.65e-11<br>chromatin silencing at rDNA<br>5 GO:0060964 1.42e-07<br>regulation of gene silencing by miRNA	23541921<br>(38 refs)	Sponge lincRNA-RoR regulates Oct4, Nanog, and Sox2 in human embryonic stem cells

1) Case numbers according to Table S1.

2) The following GO terms were predicted by lncFunTK: GO:0000122 negative regulation of transcription from RNA polymerase II promoter; GO:0006366 transcription from RNA polymerase II promoter; GO:0007186 G-protein coupled receptor signaling pathway; GO:0045944 positive regulation of transcription from RNA polymerase II promoter.

3) Selected predicted GO terms, either the top two terms, or terms with clear relevance to literature data.

4) PubMed IDs associated with the gene. Where there seems to be a clear link between literature and prediction (as reflected in the comments), this is mainly linked to the top PubMed ID in each case.
